# Line Clipping in 2D: Overview, Techniques and Algorithms

**DOI:** 10.3390/jimaging8100286

**Published:** 2022-10-17

**Authors:** Dimitrios Matthes, Vasileios Drakopoulos

**Affiliations:** Department of Computer Science and Biomedical Informatics, University of Thessaly, 35131 Lamia, Greece

**Keywords:** computer graphics, geometry, intersection algorithms, line clipping, polygon clipping

## Abstract

Clipping, as a fundamental process in computer graphics, displays only the part of a scene which is needed to be displayed and rejects all others. In two dimensions, the clipping process can be applied to a variety of geometric primitives such as points, lines, polygons or curves. A line-clipping algorithm processes each line in a scene through a series of tests and intersection calculations to determine whether the entire line or any part of it is to be saved. It also calculates the intersection position of a line with the window edges so its major goal is to minimize these calculations. This article surveys important techniques and algorithms for line-clipping in 2D but it also includes some of the latest research made by the authors. The survey criteria include evaluation of all line-clipping algorithms against a rectangular window, line clipping versus polygon clipping, and our line clipping against a convex polygon, as well as all line-clipping algorithms against a convex polygon algorithm.

## 1. Introduction

**Figure 1:**
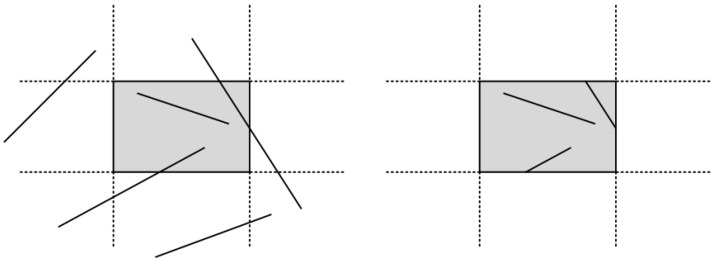
Clipping window before (**left**) and after (**right**) line clipping.

In computer graphics, any procedure that eliminates those portions of a picture that are either inside or outside a specified region of space is referred to as a *clipping algorithm* or simply *clipping*. The region against which an object is to be clipped is called a *clipping object*. In two-dimensional clipping, if the clipping object is an axis-aligned rectangular parallelogram, it is often called the *clipping window* or *clip window*. Sometimes the clipping window is alluded to as the *world window* or the *viewing window* [1]. Usually, a clipping window is a rectangle in standard position, although we could use any shape for a clipping application, e.g., a convex polygon or a concave polygonal boundary [2]. For a three-dimensional scene, the clipping area is called *clipping volume*. The process of removing lines or portions of lines outside an area of interest is called *line clipping*. Usually, any line or part of it outside the viewing area is unnecessary and is removed; see Figure 1.

The line-clipping process uses mathematical equations or formulas for removing the unnecessary parts of the line. The programmer draws only the part of the line which is visible and inside the desired region by using, for example, the slope-intercept form y=ax+b, where *a* is the slope or gradient of the line, *b* is the *y*-intercept of the line and *x* is the independent variable of the function y=f(x) or just the vector equation. Most of the time, clipping is applied to extract a part of a scene or a world, for creating new object boundaries, for managing multiple areas of objects inside a window, and so on.

There are four fundamental algorithms for line clipping: Cohen–Sutherland, Cyrus–Beck [3], Liang–Barsky [4] and Nicholl–Lee–Nicholl [5]. Over the years, other algorithms for line clipping emerged, such as Midpoint Subdivision, Fast Clipping [6], Skala ’93 [7], Skala ’94 [8], Skala 2005 [9], S-Clip E2 [10], Ray [11], Andreev and Sofianska [12], Day [13], Rappoport [14], Dimri [15], but many of them are variations of the first ones. In general, the existing line-clipping algorithms can be classified into three types: the encoding approach (with the Cohen–Sutherland algorithm as a representative), the parametric approach (with the Cyrus–Beck and Liang–Barsky algorithms as representatives) and the Midpoint Subdivision algorithms.

A different algorithm for clipping line segments by a rectangular window on a rectangular coordinate system is presented in [16,17]. For the line segments that cannot be identified as completely inside or outside the window by simple tests, this algorithm applies affine transformations (the shearing transformations) to the line segments and the window and changes the slopes of the line segments and the shape of the window. A mathematical model for evaluating intersection points, and thereby clipping lines that decently rely on integral calculations, has been proposed in [18]. A fairly full picture of the relevant literature is presented in [19,20,21] until the day of their publication.

The present article aims to present an overview of the most common, as well as of the lesser-known, algorithms and techniques for clipping a line against a rectangular area or a convex polygon in a two-dimensional space. Moreover, two new algorithms were presented; one for clipping a line against a rectangular clipping window as well as a convex clipping region. These new algorithms overcome many disadvantages of the common ones.

## 2. Fundamental Line-Clipping Algorithms

### 2.1. Cohen–Sutherland

**Figure 2:**
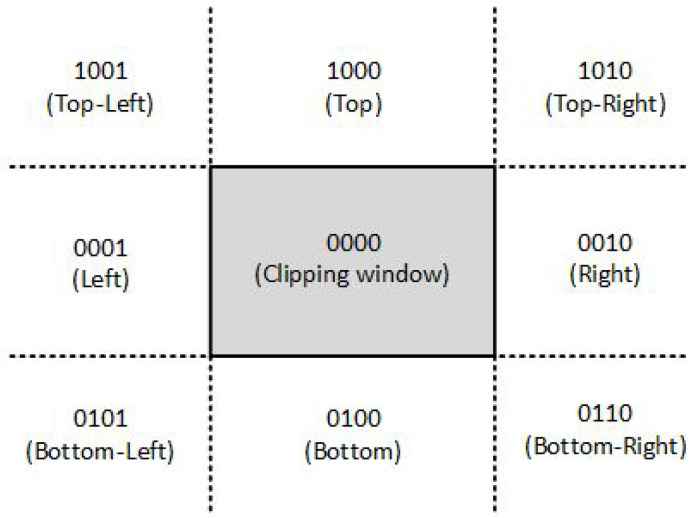
The codes for the nine regions of the Cohen-Sutherland algorithm in the two-dimensional space.

Intersection algorithms with a rectangular area (window), well known as line clipping or as line segment clipping algorithms, were developed and used for a flight simulator project led by Cohen [22] in 1969. Efficient coding of a line segment position coding leading to significant computational reduction was introduced in [23] and patented in [24]. It is considered to be one of the first line-clipping algorithms in computer graphics history and variations of this method are widely used. Processing time is reduced by performing more tests before proceeding to the intersection calculations. The two-dimensional space in which the line resides is divided into nine regions, eight “outside” regions and one “inside” region, and to each line endpoint is assigned a four-digit binary value called the *region code* (Figure 2). Each bit of the region code is used to indicate whether the point is inside or outside a region out of the nine ones [25].

**Figure 3:**
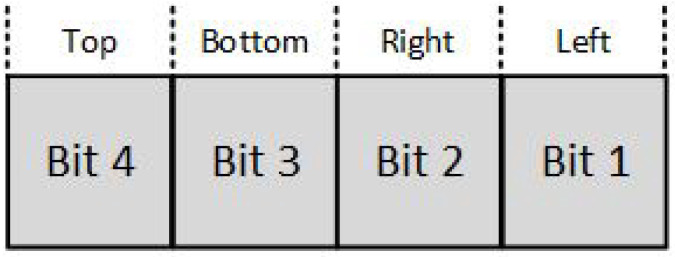
Cohen–Sutherland endpoint region code.

The region code for each line endpoint is applied using the following method: The window edges are referenced in any order with the bit positions numbered one through four from the Least Significant Bit (LSB) to the Most Significant Bit (MSB) (see Figure 3). A value of one in any of these bits indicates that the endpoint is outside the clipping window. Likewise, a value of zero to all of these bits indicates that the endpoint is inside or on the clipping window. The region code is also known as *outcode*.

At first, the algorithm assigns an outcode to each line endpoint. Next, it determines if the line is inside the clipping window. A line that is inside the clipping window has both endpoint outcodes equal to 0000. Next, the algorithm checks if there are two bits with the value one in the same bit position for each endpoint. If this is true then the line is being rejected as it is completely outside the clipping window. To check this, a logical AND is performed and if the result is not 0000 then the line is neither inside nor crossing into the clipping window, so it can be eliminated. If the result is 0000 then the line may cross into the clipping window or it could intersect one or more boundaries without entering the clipping window. It is considered for clipping and the intersection points with the clipping-window edges have to be found. Each edge is being checked against the line and those portions of the line that are outside each boundary are being clipped.

Since the algorithm checks each line endpoint that is outside the clipping window against each boundary in order to find the corresponding coordinates of the intersection, the line endpoint coordinates may be successively replaced by the corresponding intersection coordinates until meeting the correct ones.

### 2.2. Cyrus–Beck

The Cyrus–Beck (CB) algorithm was published in 1978 and is based on a parametric representation of the line segments [3]:

$$P\left(t\right)=P_{0}+t\cdot \left(P_{1}-P_{0}\right).\;0\leq t\leq 1$$

**Figure 4:**
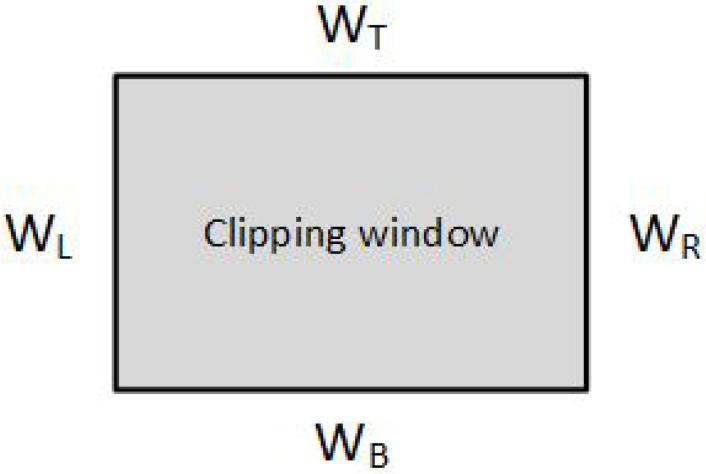
Typical clipping boundaries.

The algorithm can be applied to a typical rectangular clipping window (Figure 4) or any other convex polygon, unlike the Cohen–Sutherland which uses only rectangular clipping windows. The number of sides is not important, although it does affect performance.

**Figure 5:**
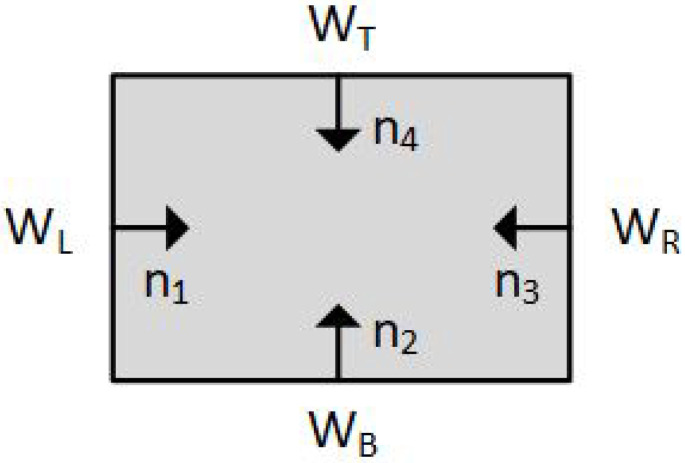
Inward normals of a rectangular clipping window.

For any convex clipping window, the *inward normals* have to be calculated. These inward normals are vectors perpendicular to each window edge. For a typical rectangular clipping window, only four unique inward normals exist and all other normals are mathematically equivalent to these four (Figure 5). The inward normals can be used for finding the intersection points between the line segment and the edges.

**Figure 6:**
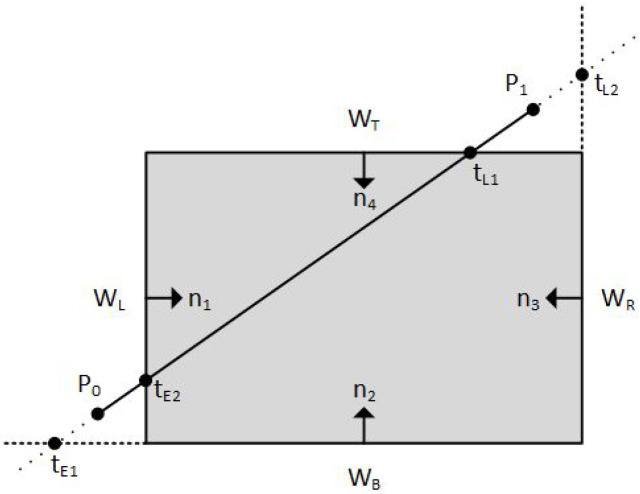
Classifying intersection points either as *Potentially Entering (PE)* or *Potentially Leaving (PL)* and calculating their *t* parameter, respectively.

The number of intersections of a straight line with the boundaries of the convex clipping window is equal to the number of the edges of the clipping window. The algorithm calculates all the intersections and classifies them either as *Potentially Entering (PE)* or *Potentially Leaving (PL)* relative to the clipping window. A PE intersection means that as we are moving on the line, the clipping window is “in the front” and we are going to “enter into” it. Likewise, a PL intersection means that as we are moving on the line, the clipping window is “in the back” and we are going to “leave out” of it. In practice, the algorithm checks each intersection if it is a PE or a PL point and calculates its $t_{E}$ or $t_{L}$ value, respectively, and forms two *t* groups; one group with all $t_{E}$ values and one group with all $t_{L}$ values (see Figure 6). The *t* values that are either less than zero or greater than one are rejected.

Since the line segment intersects the boundaries of the clipping window in, at most, two places, the algorithm selects only one $t_{E}$ value out of all the $t_{E}$ values which is the maximum one (closer to the clipping window). Similarly, it selects only one $t_{L}$ value out of all the $t_{L}$ values which is the minimum one.

**Figure 7:**
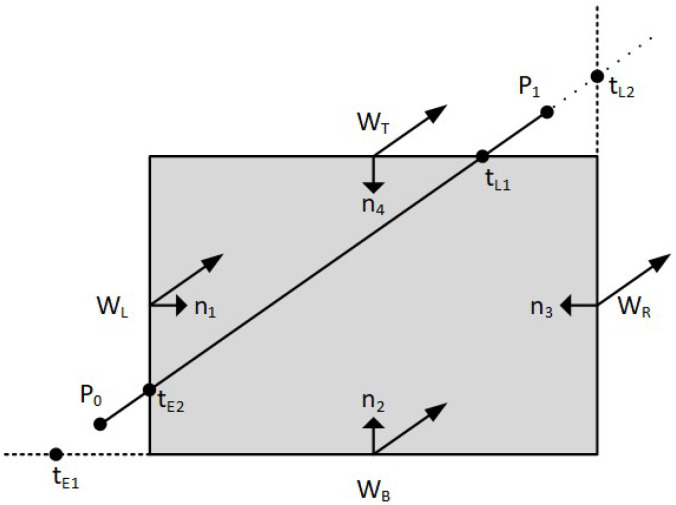
Comparing each inward normal with vector $\vec{P_{0}P_{1}}$.

The classification of the intersection points as PE or PL is performed by comparing the angle between each inward normal and the vector $\vec{P_{0}P_{1}}$. If the angle is less than 90° then the intersection point is a PE. If the angle is greater than 90° then the intersection point is a PL (see Figure 7).

Cyrus–Beck is a generalized line clipping algorithm that was designed to be more efficient than other clipping algorithms, such as the Cohen–Sutherland. It uses repetitive clipping, is very stable and its performance is nearly independent of factors such as the geometrical distribution of clipped primitives [9]. Since the algorithm computes the intersection for each edge, its complexity is O(N), where *N* is the number of edges. In most cases, it clips only once or twice unlike Cohen–Sutherland where the lines are clipped about four times. The algorithm can be easily modified to also clip three-dimensional lines.

### 2.3. Liang–Barsky

You-Dong Liang and Brian Barsky were based on Cyrus–Beck and developed an even faster algorithm for line clipping. Their algorithm uses the parametric line equations and does more line testing before proceeding to the intersection calculations. By using the parametric equation of the line, it solves four inequalities to find the range of the parameter for which the line is in the viewport [4].

**Figure 8:**
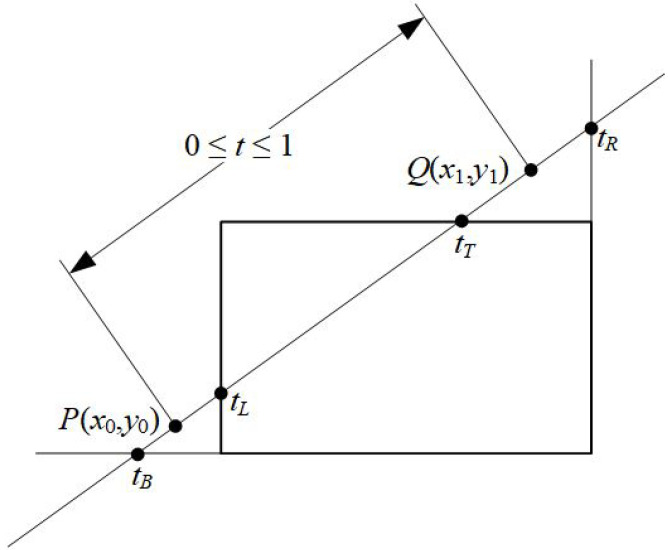
Defining the line for clipping with the Liang–Barsky algorithm.

For a line segment with endpoints $P(x_{0},y_{0})$ and $Q(x_{1},y_{1})$, we can describe the line with the parametric form

$$\begin{array}{cc} x=x_{0}+t\Delta x & \;\\ y=y_{0}+t\Delta y & \;0\leq t\leq 1 \end{array}$$

where $\Delta x=x_{1}-x_{0}$ and $\Delta y=y_{1}-y_{0}$ (see Figure 8).

Since the clipped line segment lies in the clipping window, the above parametric line equations can be combined with the following conditions

$$\begin{array}{c} x_{\min}\leq x_{0}+t\Delta x\leq x_{\max}\\ y_{\min}\leq y_{0}+t\Delta y\leq y_{\max}. \end{array}$$

These conditions can also be written as

$$\begin{array}{c} -t\Delta x\leq x_{0}-x_{\min}\\ t\Delta x\leq x_{\max}-x_{0}\\ -t\Delta y\leq y_{0}-y_{\min}\\ t\Delta y\leq y_{\max}-y_{0} \end{array}$$

or, simply, as

$$t\cdot p_{k}\leq q_{k}\;k=1,2,3,4$$

where k=1,2,3, and 4 correspond to the left, right, bottom, and top boundaries, respectively, and parameters *p* and *q* are defined as

$$\begin{array}{cc} p_{1}=-\Delta x, & \;q_{1}=x_{0}-x_{\min}\\ p_{2}=\Delta x, & \;q_{2}=x_{\max}-x_{0}\\ p_{3}=-\Delta y, & \;q_{3}=y_{0}-y_{\min}\\ p_{4}=\Delta y, & \;q_{4}=y_{\max}-y_{0}. \end{array}$$

From the aboves we can draw the following conclusions:

− If the line has $p_{k}=0$ then it is parallel to the corresponding clipping-window edge.
− If the line has $p_{k}=0$ and $q_{k}<0$ then it is completely outside and is being rejected.
− If $p_{k}>0$, the line proceeds from the inside to the outside.
− If $p_{k}<0$, the line proceeds from the outside to the inside.

For a nonzero value of $p_{k}$, $r_{k}=\frac{q_{k}}{p_{k}}$ gives value *t* for the intersection point of the line and the window edge. There are two (out of four) actual intersections with values $t_{1}$ and $t_{2}$ value, respectively. For $t_{1}$, the algorithm calculates all *t* values for which $p_{k}<0$ (line proceeds from the outside to the inside) and assigns to it the maximum one. For $t_{2}$, the algorithm calculates all *t* values for which $p_{k}>0$ (line proceeds from the inside to the outside) and assigns to it the minimum one. If $t_{1}>t_{2}$, the line is completely outside the clipping window and it can be rejected. Otherwise, the endpoints of the clipped line are calculated from the two values of parameter *t*.

In general, the Liang–Barsky algorithm is more efficient than the Cohen–Sutherland line-clipping algorithm as well as the Cyrus–Beck. It uses floating-point arithmetic for finding the appropriate endpoints with, at most, four computations [21]. In contrast, the Cohen and Sutherland algorithm can calculate intersections repeatedly even if the line is completely outside the clipping window. Moreover, the Cohen–Sutherland intersection calculation requires both a division and a multiplication. The algorithm can be easily modified to clip lines in a three-dimensional space.

### 2.4. Nicholl–Lee–Nicholl

The Nicholl–Lee–Nicholl (NLN) is an algorithm that was created in 1987 by Tina M. Nicholl, D.T. Lee and Robin A. Nicholl. Its main characteristic is that it avoids a lot of computations of the intersection points. The creators claim that its performance is better than other algorithms, e.g., the Cohen–Sutherland and Liang–Barsky, since it carries out more region testing and it performs fewer comparisons and divisions. Unfortunately, while Cohen–Sutherland and Liang–Barsky can easily extend to three dimensions, NLN clipping is limited only to two dimensions.

**Figure 9:**
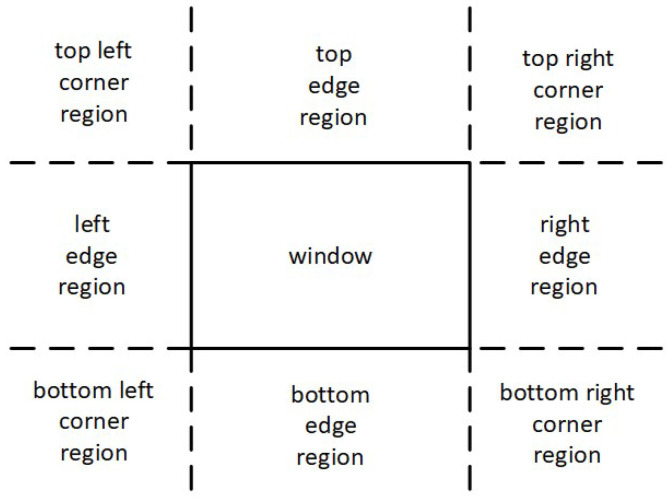
NLN divides screen space into nine regions, like CS.

**Figure 10:**
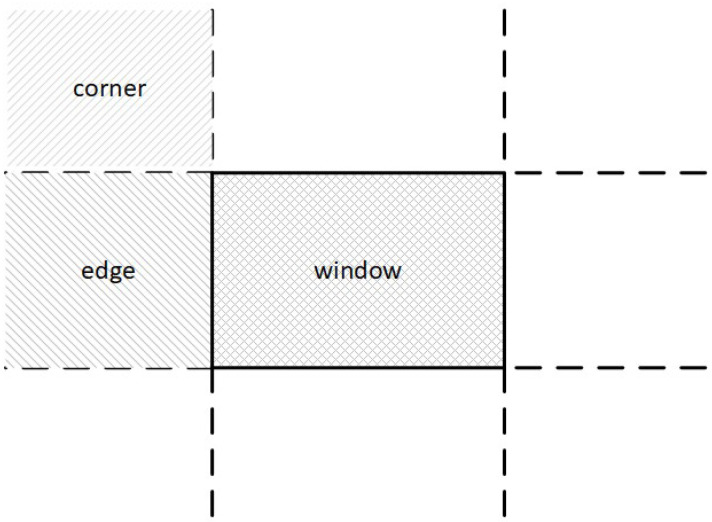
NLN uses only three out of nine regions namely window, edge and corner.

As already mentioned, Cohen–Sutherland divides the screen space into nine regions. For avoiding unnecessary checks and calculations, NLN adopts a similar scheme (Figure 9) but it uses only three out of the nine ones; the top left corner region, the left edge region and the window region (Figure 10).

**Figure 11:**
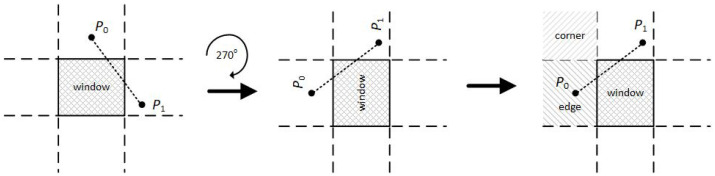
Applying 270 degrees clockwise rotation for moving the first line endpoint into the edge region.

The first endpoint of the line has to be in one of these three regions. If it lies on any of the other six, then it can be moved to one of these three regions using geometrical transformations. The second endpoint of the line is taken into account later. For example, if a line with endpoints $P_{0}$ and $P_{1}$ has the first endpoint directly above the clipping window then it can be translated into the left edge region using a 270 degrees clockwise rotation (see Figure 11).

All the available geometrical transformations are:

- 90° clockwise rotation about the origin.
- 180° clockwise rotation about the origin.
- 270° clockwise rotation about the origin.
- Reflection about the line x=−y.
- Reflection about the x−axis.

Obviously, these geometrical transformations should also be applied to the boundaries of the clipping window as well as the line endpoints.

**Figure 12:**
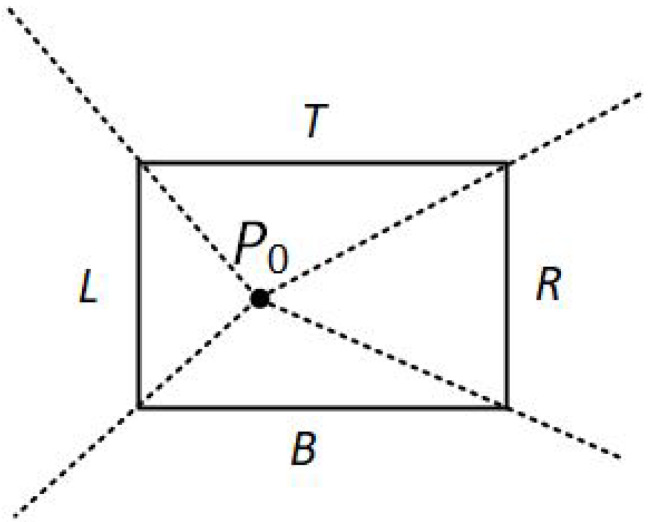
The four regions when $P_{0}$ is inside the clipping window and $P_{1}$ is outside.

**Figure 13:**
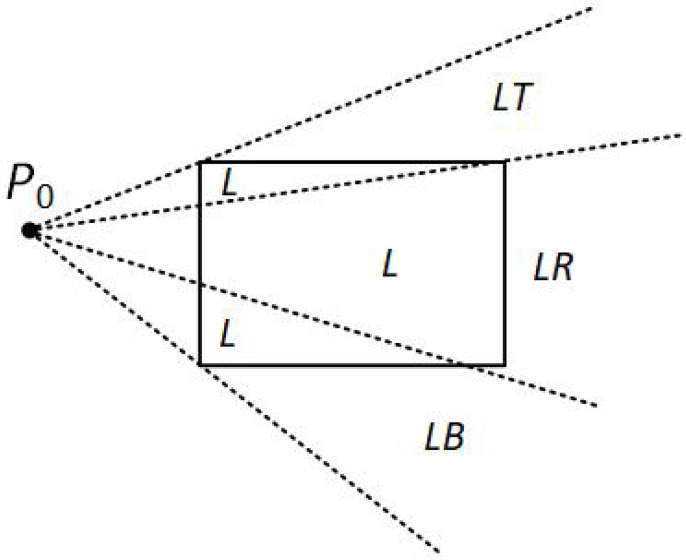
The four clipping regions when $P_{0}$ is on the edge region.

**Figure 14:**
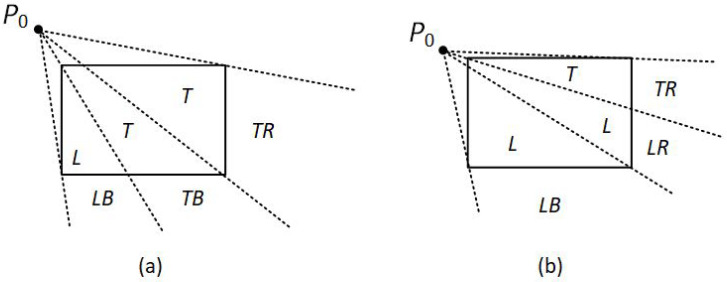
The two possible sets of clipping regions used in the NLN algorithm when $P_{0}$ is (**a**) above and (**b**) to left of the clipping window.

Assuming that $P_{0}$ and $P_{1}$ are not simultaneously inside the clipping window, the algorithm divides again the screen space into regions. The new regions are based on the position of the first line endpoint ($P_{0}$). The boundaries of the new regions are semi-infinite line segments that start at the position of $P_{0}$ and pass through each clipping window corner. Since the algorithm uses three regions, there are three main cases:

1. $P_{0}$ is inside the clipping window and $P_{1}$ outside.
  The algorithm sets up four regions (L, T, R, B) as in Figure 12. Then, depending on which one of the four regions contains $P_{1}$, it computes the line-intersection position with the corresponding window boundary.
2. $P_{0}$ is on the edge region and $P_{1}$ is outside the clipping window.
  The algorithm sets up four regions labeled L, LT, LR, and LB as in Figure 13. These four regions again determine a unique clipping-window edge for the line segment, relative to the position of $P_{1}$. For instance, if $P_{1}$ is in any one of the three regions labeled L, the algorithm clips the line at the left window border and draws the line segment from this intersection point to $P_{1}$. If $P_{1}$ is in region LT, it draws the line segment from the left window boundary to the top boundary. Likewise, the same logic applies to regions LR and LB. However, if $P_{1}$ is not in any of these four regions, the line is clipped entirely.
3. $P_{0}$ is on the corner region and $P_{1}$ is outside the clipping window.
  When $P_{0}$ is to the corner region, the algorithm uses one of the two sets as shown in Figure 14. The selection of (a) or (b) depends on the position of $P_{0}$ within the corner region. When $P_{0}$ is closer to the left clipping boundary of the window, the algorithm uses the regions in (a) of this figure but when $P_{0}$ is closer to the top clipping boundary of the window, it uses the regions in (b). If $P_{1}$ is in one of the regions T, L, TR, TB, LR, or LB, this determines a unique clipping-window border for the intersection calculations, otherwise, the entire line is rejected.

**Figure 15:**
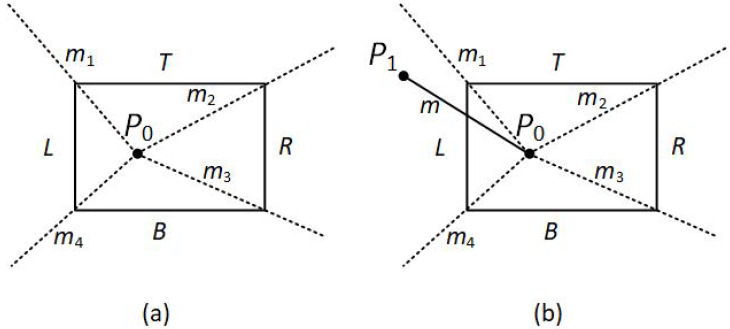
(**a**) $m_{1},\;m_{2},\;m_{3},\;m_{4}$ are the slopes of the line segments formed between $P_{0}$ and L, T, R, B, boundaries respectively. (**b**) *m* is the slope of the line segment $P_{0}P_{1}$.

To determine the region in which $P_{1}$ is located, NLN compares the slope of the line segment against the slopes of the new boundaries. For example, if $P_{0}$ is inside the clipping window and $P_{1}$ is outside, *m* is the slope of the line segment $P_{0}P_{1}$ and $m_{1},\;m_{2},\;m_{3},\;m_{4}$ are the slopes of the boundaries L, T, R, B, respectively (see Figure 15a), then according to the following conditions, $P_{1}$ is:

- $m_{1}<m<m_{2}\to P_{1}$ is above the clipping window.
- $m_{2}<m<m_{3}\to P_{1}$ is on the right of the clipping window.
- $m_{3}<m<m_{4}\to P_{1}$ is below the clipping window.
- $m_{4}<m<m_{1}\to P_{1}$ is on the left of the clipping window.

Suppose that $P_{1}$ is on the left region (see Figure 15b). From the parametric equations

$$\begin{array}{c} x=x_{0}+\left(x_{1}-x_{0}\right)u\\ y=y_{0}+\left(y_{1}-y_{0}\right)u \end{array}$$

an x-intersection position on the left window boundary is calculated as $x=x_{L}$, with $u=\left(x_{L}-x_{0}\right)/\left(x_{1}-x_{0}\right)$, so that the y-intersection position is

$$y=y_{0}+\frac{y_{1}-y_{0}}{x_{1}-x_{0}}\left(x_{L}-x_{0}\right)$$

## 3. Common Line-Clipping Algorithms

### 3.1. Midpoint Subdivision

Midpoint Subdivision (MS) is an extension of the Cohen–Sutherland algorithm and follows the divide and conquer strategy. It is mainly used to compute the visible areas of lines that are present in the clipping window. It follows the principle of bisecting the line into equal halves numerous times. The algorithm is not efficient unless it is implemented in hardware. Moreover, the Cohen–Sutherland line clipping algorithm requires the calculation of the intersection of the line with the window edge. These calculations can be avoided by repetitively subdividing the line at its midpoint.

**Figure 16:**
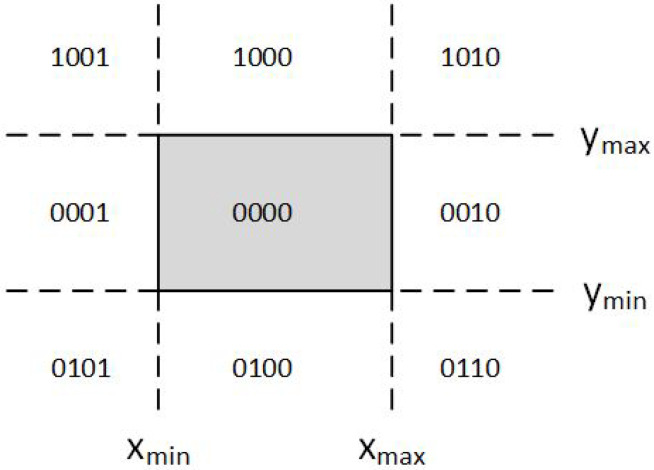
Outcodes for the Midpoint Subdivision algorithm.

At first, the algorithm categorizes the endpoints of the line segment and assigns a four-bit region code to each one like Cohen–Sutherland does. The code, also known as outcode, is determined according to which of the following nine regions of the plane the endpoint lies in (see Figure 16).

**Figure 17:**
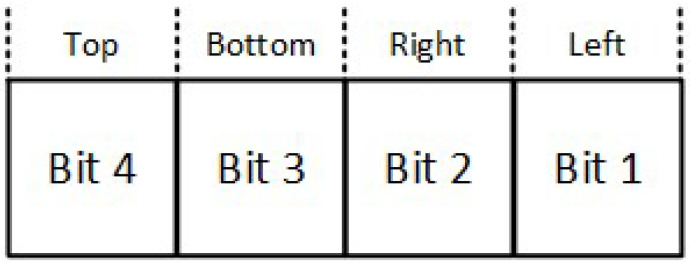
Outcode bits for the Midpoint Subdivision algorithm.

Starting from the Least Significant Bit (LSB), each bit represents one region; left, right, bottom, top (see Figure 17). If a line endpoint is inside that region then the corresponding bit is set to true (1) or otherwise false (0).

There are three possible cases for any given line.

1. **Totally visible**: If the outcode of both line segment endpoints is 0000 then the line segment is inside the clipping window and it is completely visible.
2. **Totally invisible**: Bitwise *AND* between the two outcodes of the line segment endpoints. If the result is not 0000 then the line endpoints share the same region and the line segment does not cross the clipping window so it is rejected.
3. **Clipping candidate**: If the line is in neither Category 1 nor Category 2 then it is partially visible and has to be subdivided into two equal parts. The visibility tests are then applied to each half. This subdivision process is repeated until we obtain completely visible and completely invisible line segments.

### 3.2. Skala 2005

The Skala 2005 (SKA05) performs line clipping against an ordinary rectangle clipping window as well as against any convex polygon. It does not require a division operation and uses homogeneous coordinates for input and output point representation. According to professor Vaclav Skala, its creator, it takes advantage of operations supported by vector–vector hardware [9].

**Figure 18:**
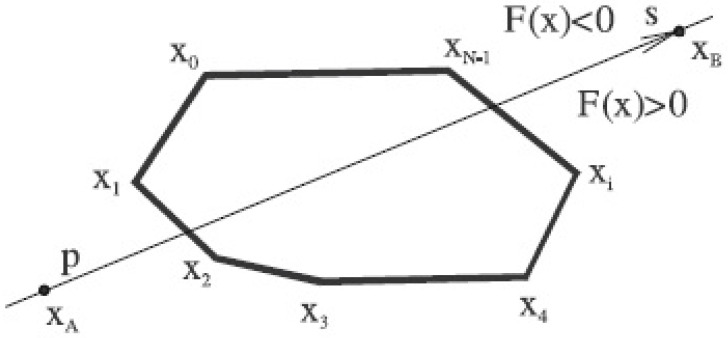
Classification of each vertex.

The algorithm assumes a convex polygon P and a line p given as F(x)=ax+by+c=0. As the line p subdivides the space into two half-spaces, the function F(x) is being evaluated for each vertex of the convex polygon (see Figure 18).

For the $C_{i}$ vertex, the classification is performed like this; the digit 1 means that the vertex is left to the line and the digit 0 means that it is right to the line. A sequence of 0 and 1 is formed and by the alternations from digit 0 to digit 1 and vice versa we can understand which edges of the polygon are being intersected by the line. These edges are marked as TAB1 and TAB2. For every possible combination of the TAB1 and TAB2, there is a binary value known as the MASK which is used in the next steps of the algorithm to determine which endpoints of the line are inside or outside the clipping area. No matter how many vertices, the TAB1 and TAB2 values are two, so the following table is used as an index to the TAB1-TAB2-MASK values; see Table 1).

**Figure 19:**
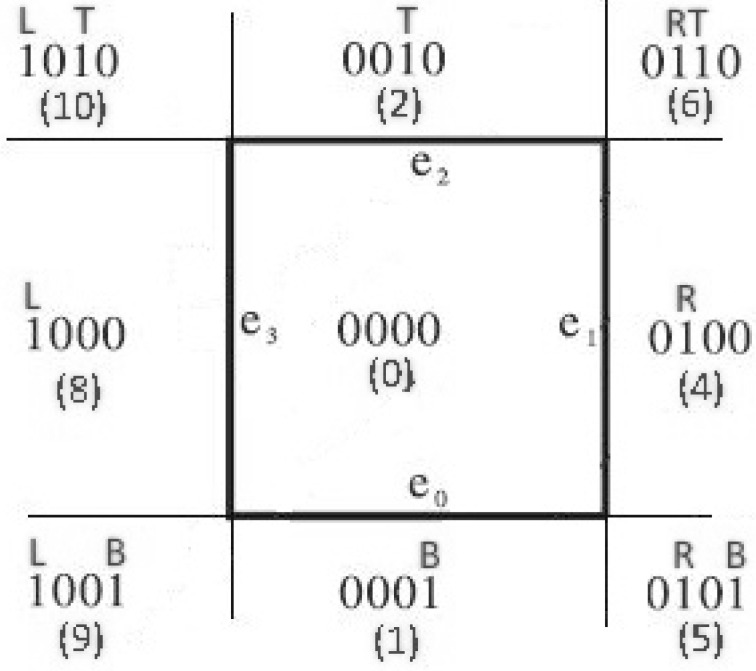
Screen is divided into 9 regions.

Having found the intersections between the line and the edges that the TAB1 and TAB2 values indicate, the algorithm classifies the endpoints of the line segment in a similar way to how Cohen–Sutherland does. It divides the screen into nine regions with each region having a unique binary number with four digits, known as the *outcode*. The four digits of each outcode represent the regions LEFT-RIGHT-TOP-BOTTOM, respectively, which means that the MSB (Most Significant Bit) represents the LEFT region, the next bit represents the RIGHT region, the next bit represents the TOP region, and finally, the LSB (Least Significant Bit) represents the BOTTOM region (see Figure 19).

The outcode is calculated for each endpoint using the function “CODE” of Algorithm 1.
**Algorithm 1:** Function to determine the outcode of the endpoints.**function** CODE(*x*);**begin**        *c* := [0000];        **if** *x* < $x_{\min}$**then***c* := [1000]               **else if** *x* > $x_{\max}$**then***c* := [0100];        **if** *y* < $y_{\min}$**then***c* := *c* **lor** [1001]               **else if** *y* > $y_{\max}$**then***c* := *c* **lor** [0010];        CODE := *c***end** [CODE];

   The outcodes show which endpoints are inside or outside the clipping area. In some cases, the MASK is additionally used in order to decide which of the two intersection points has to be used in the clipping process. Finally, the clipped line is drawn.

The speed of the algorithm varies. For a standard rectangle clipping window, the algorithm may use a predefined TAB-MASK table in order to quickly calculate the TAB1 and TAB2 values so its speed is high. However, when the clipping area is a convex polygon the speed decreases for two reasons. The first one is related to the calculations of the alternations of the 0 and 1 digits; they have to be performed “on-the-fly”, so this procedure slows down the algorithm. The second has to do with the way the algorithm works. No matter how many the vertices of the polygon, it always classifies them as “left” or “right” to the line and then it calculates the two intersections. After that and by using these two intersections, it forms a rectangle clipping area and re-classifies as “left” or “right” the vertices of the rectangle. Then, it follows the procedure that was mentioned before, which is to calculate the outcodes of the endpoints of the line and then perform clipping. However, this “double classification” of the vertices makes the algorithm slower, something that is more obvious as the number of edges increases.

### 3.3. S-Clip E2

S-clip E2 (SCE2), is another clipping algorithm also made by professor Vaclav Skala in the year 2012. Technically, it is an improved Skala 2005 algorithm that is based on the principle “test first and then compute”. Unlike other algorithms, e.g., Cohen–Sutherland, it evaluates the position of the given line with respect to the corners of the clipping window [10]. The main difference between the S-clip E2 and the Skala 2005 is that there is no need to repeat the classification process for the intersection points. Suppose that the line that has to be clipped is defined by two points, *A* and *B*. Since the intersection points belong on this line, the algorithm calculates the parameter “t” of the parametric form of the line segment AB (x=a+bt,y=c+dt) for each one. Two “t” values derive (scalars), i.e., $t_{\min}$ and $t_{\max}$ and the resulting segment is determined as $<t_{\min},t_{\max}>\cap <0,1>$ which is a trivial operation. If the orientation of the clipping window is known, no ordering of “t” values is needed.

## 4. Uncommon Line-Clipping Algorithms

### 4.1. Kodituwakku–Wijeweere–Chamikara

**Figure 20:**
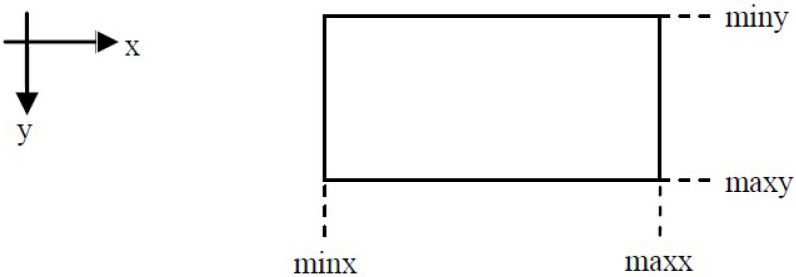
The rectangular clipping window of the KWC algorithm.

In 2013, another fast line clipping algorithm with a similar approach to the Cohen–Sutherland was introduced by Kodituwakku–Wijeweere–Chamikara [26]. The rectangular clipping window is defined by two points: (minx,miny) and (maxx,maxy) and the line is defined by two points $A(x_{0},y_{0})$ and $B(x_{1},y_{1})$; see Figure 20.

**Figure 21:**
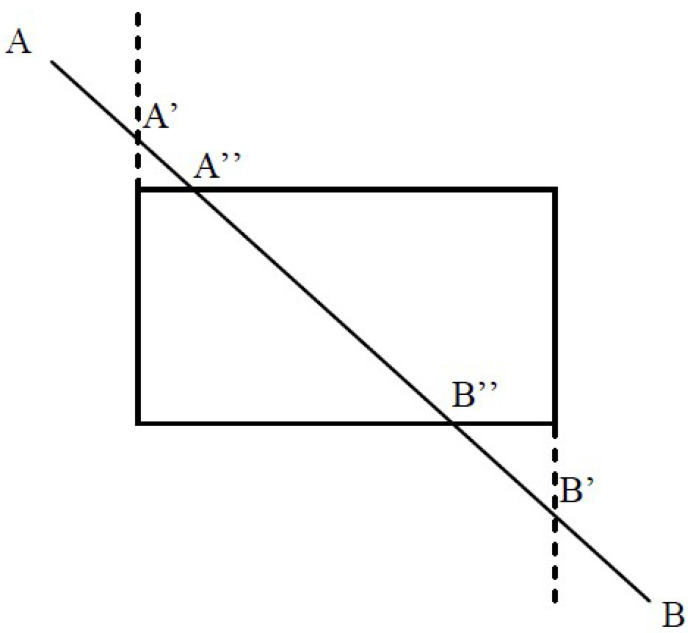
Line is intersecting the boundaries; The new line endpoints $A^{\prime \prime }$ and $B^{\prime \prime }$ have been calculated.

The coordinates of each line endpoint are checked against the boundaries of the rectangular clipping window. In case a coordinate exceeds the boundary of the clipping window then the coordinate of this boundary is used and the other coordinate is calculated by using the equation of the line in the two-dimensional space: y=m·x+c, where *m* is the slope of the line given by the formula: $m=\frac{y_{1}-y_{0}}{x_{1}-x_{0}}$; see Figure 21.

### 4.2. Matthes–Drakopoulos Line Clipping against a Rectangular Window

Each of the fundamental algorithms mentioned before has advantages and disadvantages. In 2019, Matthes and Drakopoulos introduced an efficient line-clipping algorithm [27] or [28] which aims at simplicity and speed and does only the necessary calculations in order to clip a line inside the clipping window.

**Figure 22:**
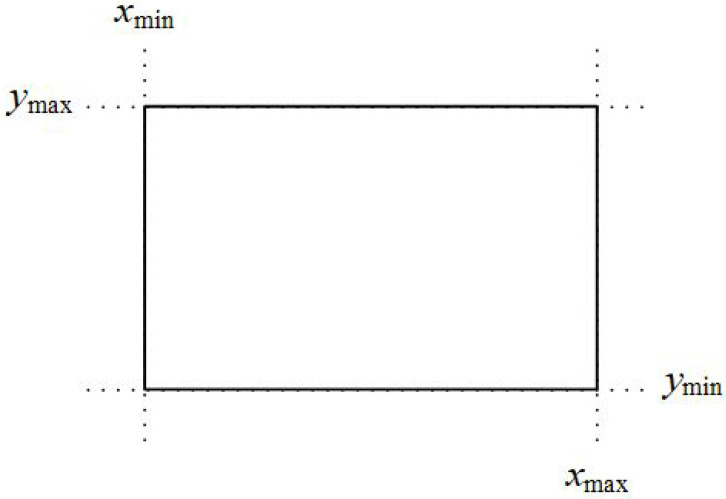
Line clipping region.

Assume that we want to clip a line segment that crosses a rectangle clipping window that is defined by the points $\left(x_{\min},y_{\max}\right)$ and $\left(x_{\max},y_{\min}\right)$. This clipping window is depicted in Figure 22.

Given two points $\left(x_{1},y_{1}\right)$ and $\left(x_{2},y_{2}\right)$ on the line that we want to clip, the slope *m* is constant and is defined by the fraction

(1) $$m=\frac{y_{2}-y_{1}}{x_{2}-x_{1}}$$

For an arbitrary point (x,y) on the line, the previous ratio can be written as

$$m=\frac{y-y_{1}}{x-x_{1}}.$$

Solving for *y*

$$y-y_{1}=m\cdot \left(x-x_{1}\right)\Leftrightarrow y=y_{1}+m\cdot \left(x-x_{1}\right).$$

By replacing *m* in this equation with Equation (1)

(2) $$y=y_{1}+\frac{y_{2}-y_{1}}{x_{2}-x_{1}}\cdot \left(x-x_{1}\right).$$

Solving for *x*, the equation becomes

(3) $$x=x_{1}+\frac{x_{2}-x_{1}}{y_{2}-y_{1}}\cdot \left(y-y_{1}\right).$$

Equations (2) and (3) are two mathematical representations of the line equation y=m·x+b and will be used later by the algorithm in order to determine the part of the line that is inside the clipping window.

Suppose that the line segment which has to be clipped is defined by the points $\left(x_{1},y_{1}\right)$ and $\left(x_{2},y_{2}\right)$.

Step 1

**Figure 23:**
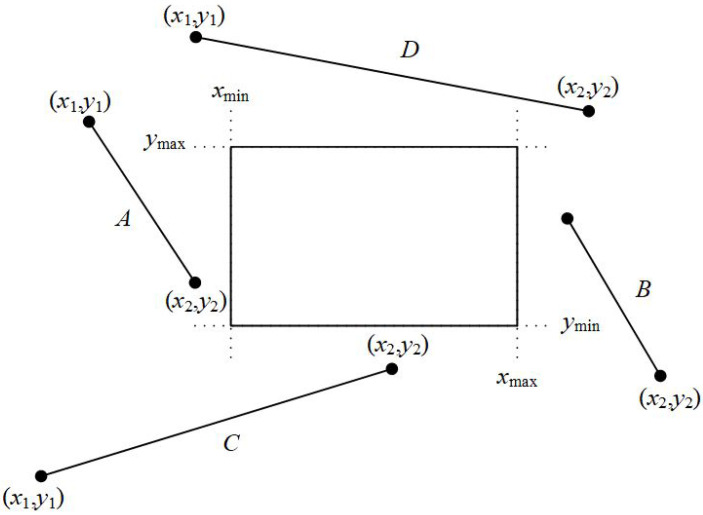
Lines A,B,C and *D* are rejected according to the first step of the algorithm.

The first step of the algorithm checks if both points are outside the line clipping window and at the same time in the same region (top, bottom, right, left). If one of the following occurs then the entire line is rejected and the algorithm draws nothing (see Figure 23):
$x_{1}<x_{\min}$ AND $x_{2}<x_{\min}$(line is left to the clipping window)$x_{1}>x_{\max}$ AND $x_{2}>x_{\max}$(line is right to the clipping window)$y_{1}<y_{\min}$ AND $y_{2}<y_{\min}$(line is under the clipping window)$y_{1}>y_{\max}$ AND $y_{2}>y_{\max}$(line is over the clipping window)

Step 2

**Figure 24:**
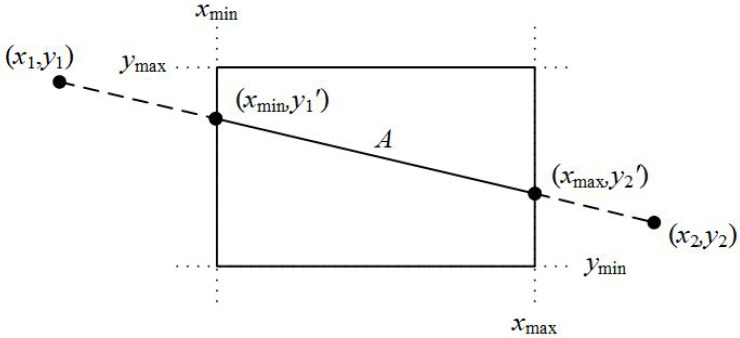
Selecting the points of the line that are inside the clipping area.

In the second step, the algorithm compares the coordinates of the two points along with the boundaries of the clipping window. It compares each of the $x_{1}$ and $x_{2}$ coordinates with the $x_{\min}$ and $x_{\max}$ boundaries, respectively, as well as each one of the $y_{1}$ and $y_{2}$ coordinates with the $y_{\min}$ and $y_{\max}$ boundaries, respectively. If any of these coordinates are out of bounds, then the specific coordinate of the boundary is used in the equation that determines the line for performing clipping (see Figure 24).

For each of the coordinates of the two points and according to Equations (2) and (3), the comparisons and changes made are:

- If $x_{i}<x_{\min}$ then
  $$x_{i}=x_{\min}$$
  $$y_{i}=y_{1}+\frac{\left(y_{2}-y_{1}\right)}{\left(x_{2}-x_{1}\right)}\cdot \left(x_{\min}-x_{1}\right)$$
- If $x_{i}>x_{\max}$ then
  $$x_{i}=x_{\max}$$
  $$y_{i}=y_{1}+\frac{\left(y_{2}-y_{1}\right)}{\left(x_{2}-x_{1}\right)}\cdot \left(x_{\max}-x_{1}\right)$$
- If $y_{i}<y_{\min}$ then
  $$y_{i}=y_{\min}$$
  $$x_{i}=x_{1}+\frac{\left(x_{2}-x_{1}\right)}{\left(y_{2}-y_{1}\right)}\cdot \left(y_{\min}-x_{1}\right)$$
- If $y_{i}>y_{\max}$ then
  $$y_{i}=y_{\max}$$
  $$x_{i}=x_{1}+\frac{\left(x_{2}-x_{1}\right)}{\left(y_{2}-y_{1}\right)}\cdot \left(y_{\max}-x_{1}\right)$$

where i: from 1 to 2.

Note that in the above equations and when $x_{i}<x_{\min}$ or $x_{i}>x_{\max}$, division with zero will never occur because $x_{1}\neq x_{2}$ from Step 1. Likewise, when $y_{i}<y_{\min}$ or $y_{i}>y_{\max}$, division with zero will never occur because $y_{1}\neq y_{2}$ for the same reason.

Step 3

The third and final step checks if the new points, after the calculations, are inside the clipping window and if so, a line is being drawn between them.

The representation of the algorithm in pseudo-code follows:

// INPUT DATA: x1, y1, x2, y2, xmin, ymax, xmax, ymin //

if(!(x1 < xmin && x2 < xmin) && !(x1 > xmax && x2 > xmax))

if(!(y1 < ymin && y2 < ymin) && !(y1 > ymax && y2 > ymax))

{

x[0] = x1;

y[0] = y1;

x[1] = x2;

y[1] = y2;

i = 1;

do

{

if(x[i] < xmin)

{

x[i] = xmin;

y[i] = ((y2 - y1)/(x2 - x1)) * (xmin - x1) + y1;

}

else if(x[i] > xmax)

{

x[i] = xmax;

y[i] = ((y2 - y1)/(x2 - x1)) * (xmax - x1) + y1;

}

if(y[i] < ymin)

{

y[i] = ymin;

x[i] = ((x2 - x1)/(y2 - y1))*(ymin - y1) + x1;

}

else if(y[i] > ymax)

{

y[i] = ymax;

x[i] = ((x2 - x1)/(y2 - y1)) * (ymax - y1) + x1;

}

i++;

}

while(i <= 1);

if(!(x[0] < xmin && x[1] < xmin) && !(x[0] > xmax && x[1] > xmax))

draw_line(x[0], y[0], x[1], y[1]);

}

## 5. Evaluation of All Line-Clipping Algorithms against a Rectangular Window

**Figure 25:**
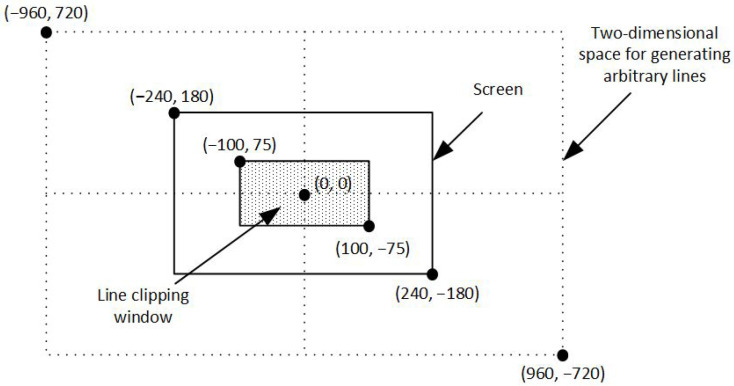
Defining the clipping window as well as the two-dimensional space for generating arbitrary lines.

For the evaluation of the line clipping algorithms, C++ programming language with OpenGL was used. The procedure of the evaluation was the following: Each algorithm created a large number of arbitrary lines in a two-dimensional space. This space was determined by the points (−960, 720) and (960, −720). The clipping window was at the center of the screen and its size was defined by the points (−100, 75) and (100, −75); that is 200 pixels width and 150 pixels height. The lines were randomly generated anywhere in the two-dimensional space and each algorithm drew only the visible part of the lines inside the clipping window (see Figure 25).

The time that each algorithm needed to clip and draw the clipped line segments was recorded in every execution. The whole process was repeated 10 times and the average time was calculated at the end. The hardware as well as the software specifications of the evaluation process were: (a) Intel Core i7-9750H @ 2.60 GHz CPU, (b) RAM 16 GB, (c) NVIDIA GeForce RTX 2070/8 GB GPU, (d) Windows 10 Pro 64 bit operating system, (e) C++ with OpenGL/Freeglut running under the Code::Blocks environment.

**Figure 26:**
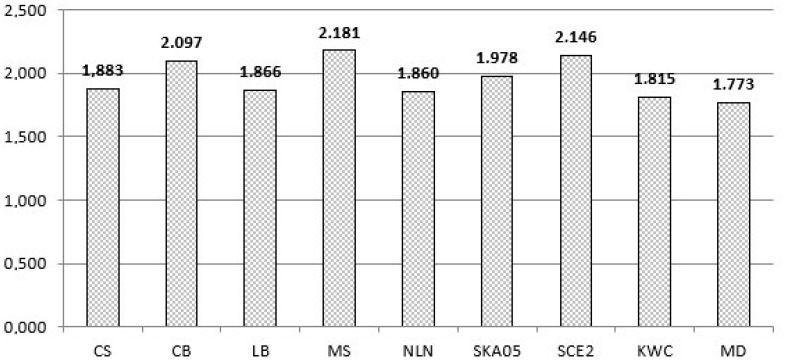
Average time of each algorithm for clipping 10 million lines (lower value → better).

Each algorithm created and clipped 10,000,000 lines in every execution. The results are shown in Table 2 and in Figure 26.

By studying the graph with the average times, we conclude that the *MD* algorithm is the fastest of all. Using the formula:

$$\frac{MD-other}{MD}\cdot 100$$

we can see how much faster in percent the *MD* algorithm is compared to the others. The next table shows these comparisons (see Table 3).

As already mentioned, each algorithm has advantages and disadvantages. The *MD* algorithm when compared with all other algorithms is not only the fastest but also the simplest. The CS algorithm has a decent performance although the oldest of all. The CB algorithm has one of the worst performances and uses advanced mathematical concepts but it can be applied to any convex polygon clipping area. Moreover, it can be easily extended to three-dimensional clipping. LB looks like CB and also uses advanced mathematical concepts but performs better. It can also be applied to three-dimensional clipping but not against a convex polygon. MS has the worst performance among all clipping algorithms due to continuous divisions. It is not easily applicable but its performance may increase if it is used with hardware clipping. The NLN algorithm has a good performance but its code is very long since it uses a large number of sub-cases and subroutines for the geometric transformations and clipping. SKA05 is a little bit complex and relatively slow due to the double classification of each vertex. SCE2 has a bad performance but it is designed to be better for clipping lines against convex polygons with more than four edges. Finally, the KWC algorithm uses a similar approach to the CS and is very fast but it uses many conditions when handling horizontal and vertical lines which makes the algorithm more complicated and slower than the *MD*.

## 6. Line Clipping vs. Polygon Clipping

**Figure 27:**
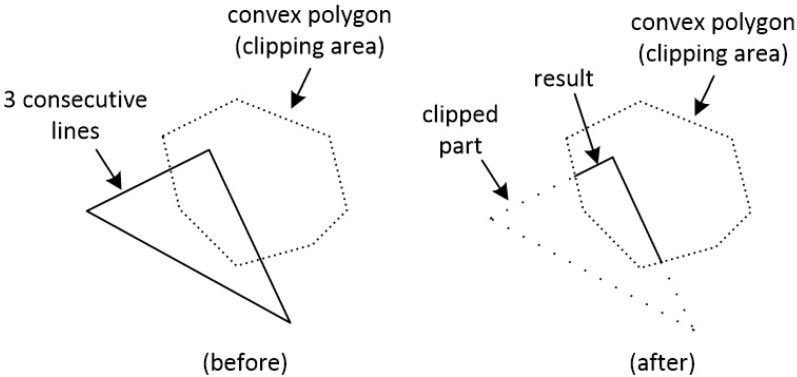
Clipping three consecutive lines that form a triangle against a convex polygon. The result is clipped lines.

The term “line clipping against a polygon” is often confused with the term “polygon clipping”. Although clipping is the main concept in both cases, these two terms describe a different behavior for each clipping procedure. Line clipping against a polygon means that one or more lines are going to be clipped one by one and the result will be just clipped lines [29]. For example, if we want to clip three consecutive lines that form a triangle against a convex polygon, the result would be only the clipped lines (see Figure 27).

**Figure 28:**
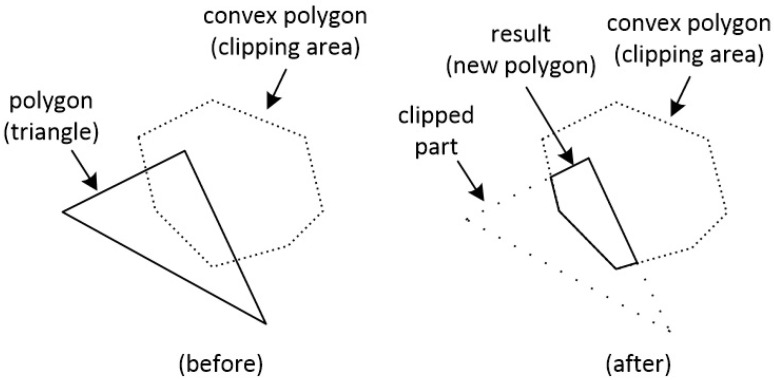
Clipping a polygon (triangle) against a polygon. The result is a new polygon.

On the other hand, polygon clipping behaves slightly differently. Clipping is applied between polygons and the result is a new polygon. So, if we want to clip a triangle against a convex polygon, the result would be a new convex polygon (see Figure 28).

There are many polygon clipping algorithms such as Weiler–Atherton [30], Sutherland–Hodgman [31], Greiner–Hormann [32], Vatti [33]. Unfortunately, these algorithms cannot work as a “line clipping against a polygon” algorithm unless they are heavily modified.

From the algorithms described before, Cyrus–Beck, Skala 2005 and S-Clip E2 can be easily modified to clip lines against a convex polygon clipping area instead of a rectangle window.

## 7. Matthes–Drakopoulos Line Clipping against a Convex Polygon

A new computation method for two-dimensional line clipping against a convex polygon clipping area is introduced. All calculations are based on a *virtual cross product* of vectors in the two-dimensional space. The algorithm, if necessary, computes only the intersection points between the line and the edges of the clipping convex polygon. The evaluation of the algorithm shows that its performance is by far better than the other relative algorithms. There is no limit to the number of vertices of the convex polygon area.

### 7.1. Mathematical Background: The Cross Product

**Figure 29:**
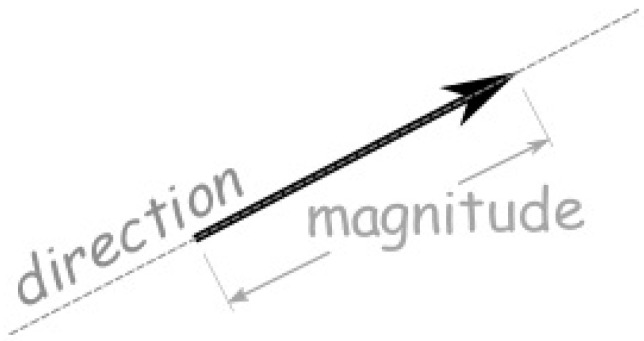
Magnitude and direction of a vector.

We know that a vector can be defined by two points and has magnitude (or length) and direction; see Figure 29.

**Figure 30:**
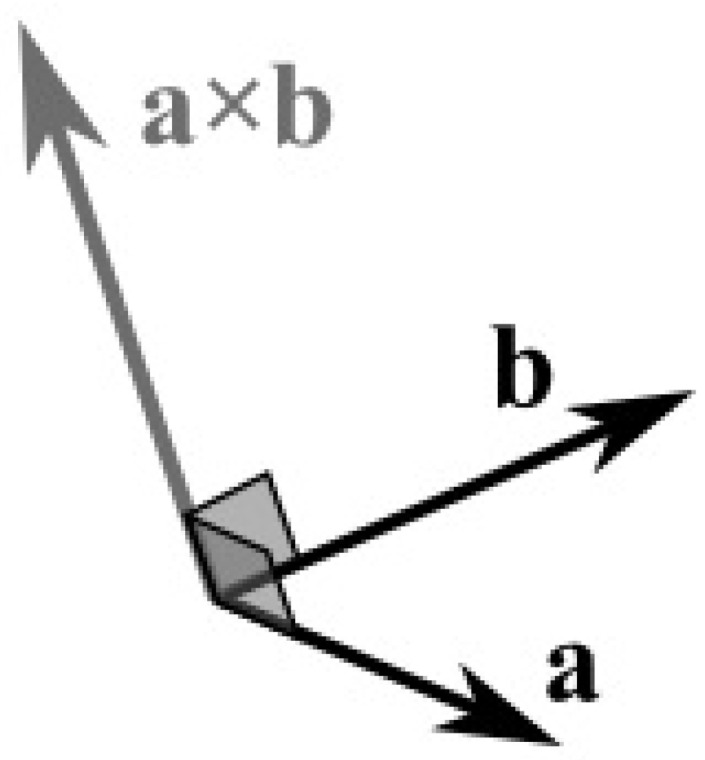
The cross product of 2 vectors is a third vector in the 3D space.

Two vectors a and b in the three-dimensional space can be multiplied using the *cross product*. The cross product of the two vectors, which is symbolized as a×b, is another vector that is at right angles to both of them (Figure 30).

**Figure 31:**
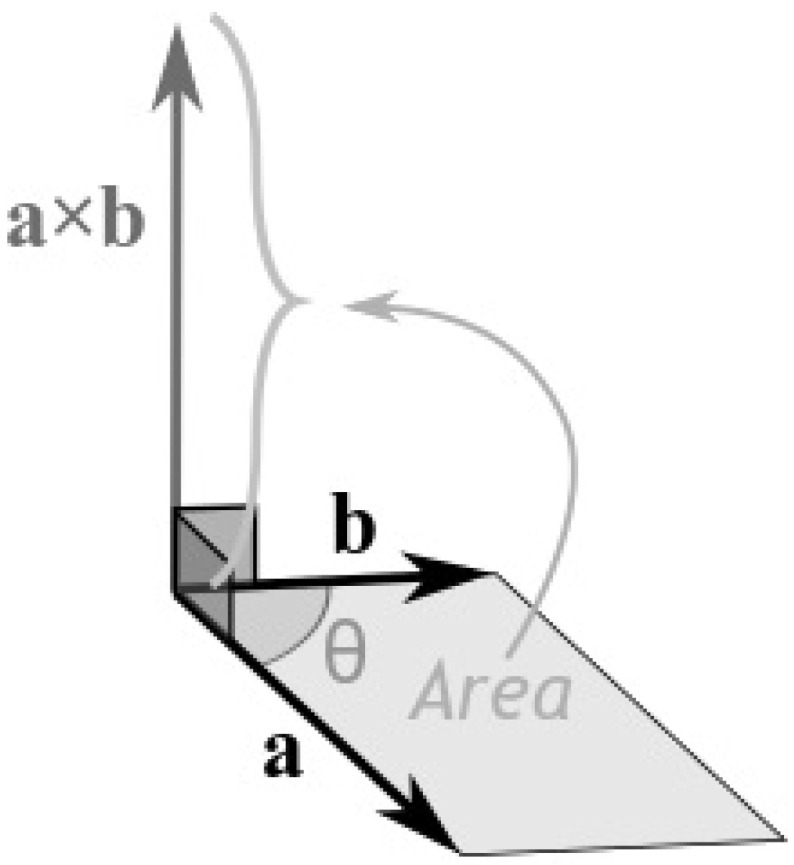
The cross product equals the area of a parallelogram with vectors a and b used as sides of the parallelogram.

The magnitude (length) of the cross product equals the area of a parallelogram with vectors a and b used as sides of the parallelogram; see Figure 31.

**Figure 32:**
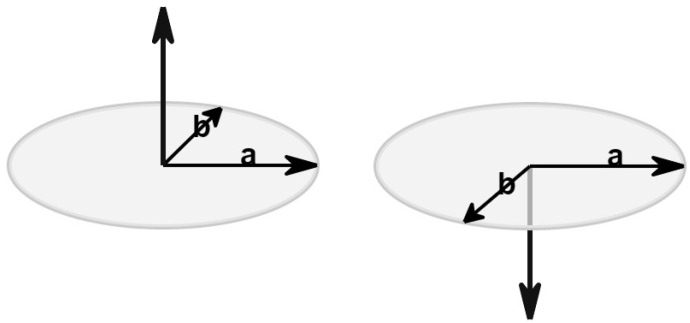
The direction changes depending on the angle of vectors a and b.

The cross product has the following characteristics:

- Has zero length, when the vectors a and b are in the same or the opposite direction.
- It reaches the maximum length when the vectors a and b are at right angles.
- The direction changes depending on the angle of vectors a and b; see Figure 32.

**Figure 33:**
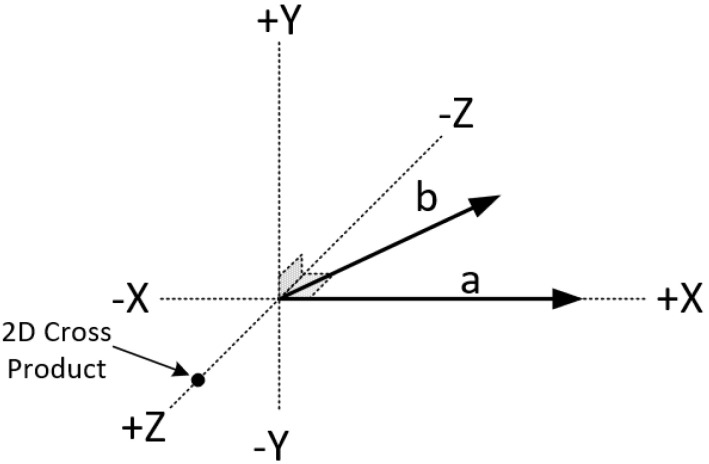
From the sign of the 2D cross product we can determine, if vector b is to the left, to the right or on vector a.

The cross product does not really exist in the two-dimensional space as the operation is not defined there. However, it is handy to assume that the cross product of two vectors in the two-dimensional space exists by assuming that these vectors are three-dimensional with their Z-coordinate set to zero. The result is a scalar (vector with only a Z-component) and it can be considered as a point perpendicular to the X-Y plane. The sign of this value represents the direction of the cross product vector in the three-dimensional space and, as a result, we can determine the orientation between the two two-dimensional vectors (see Figure 33). From now on, this virtual cross product of two-dimensional vectors will be simply referred to as “2D cross product”.

**Figure 34:**
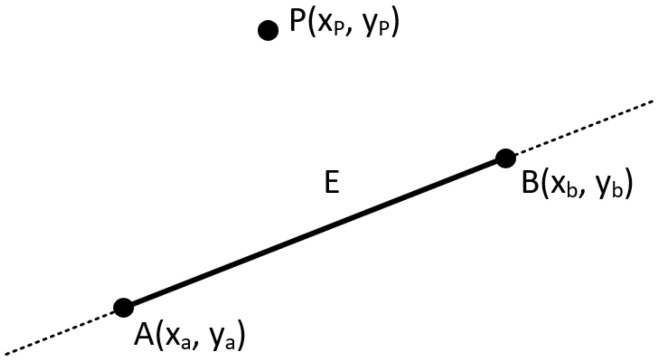
Checking if point *P* is left, right or on the line segment *E*.

**Figure 35:**
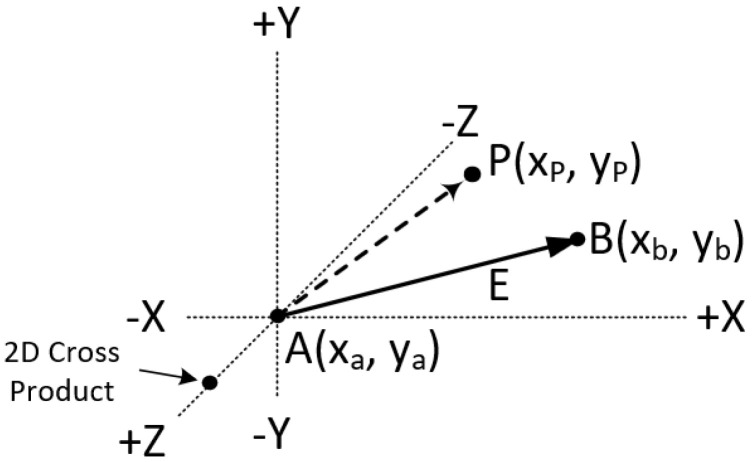
Using the 2D cross product to determine the position of the point *P* comparing to the line segment *E*.

Having said that, we can easily understand if a point $P(x_{p},y_{p})$ is to the left, to the right right or on the line segment *E* defined by the points $A(x_{a},y_{a})$ and $B(x_{b},y_{b})$ (see Figure 34 and Figure 35).

The trick is to see all these points as two vectors (vector AP and vector AB) with a common origin and then check the sign of their 2D cross product. A positive or negative value means that point *P* would be right or left to the line segment *E*, respectively, and a zero value means that the point *P* would be on it.

Let us analyze it a little bit further. Based on Figure 35, the cross product of vector $\vec{AB}$ with vector $\vec{AP}$ is:

$$\vec{AB}\times \vec{AP}=\left|\begin{array}{cc} \left(x_{b}-x_{a}\right) & \left(y_{b}-y_{a}\right)\\ \left(x_{p}-x_{a}\right) & \left(y_{p}-y_{a}\right) \end{array}\right|\Rightarrow$$

(4) $$\vec{AB}\times \vec{AP}=\left(x_{b}-x_{a}\right)\cdot \left(y_{p}-y_{a}\right)-\left(y_{b}-y_{a}\right)\cdot \left(x_{p}-x_{a}\right)$$

By using the right-hand rule, if the value of the cross product is positive then the point *P* is to the left of the vector $\vec{AB}$ and left to the line segment *E* (direction matters). Likewise, if the value is negative then point *P* is to the right. Of course, zero means that it is on the line.Having this in mind, we can create a function in pseudo-code (C++ based) that accepts three points, one arbitrary and two of the line, that returns the 2D cross product.

//  INPUT: arbitary point P, line points A & B

// OUTPUT: cross product (clockwise order)

//         > 0 : left side

//         < 0 : right side

//         = 0 : on the line

float cross_product(point P, point A, point B)

{

return (B.x - A.x) * (P.y - A.y) - (B.y - A.y) * (P.x - A.x);

}

### 7.2. Further Analysis of the Cross Product

**Figure 36:**
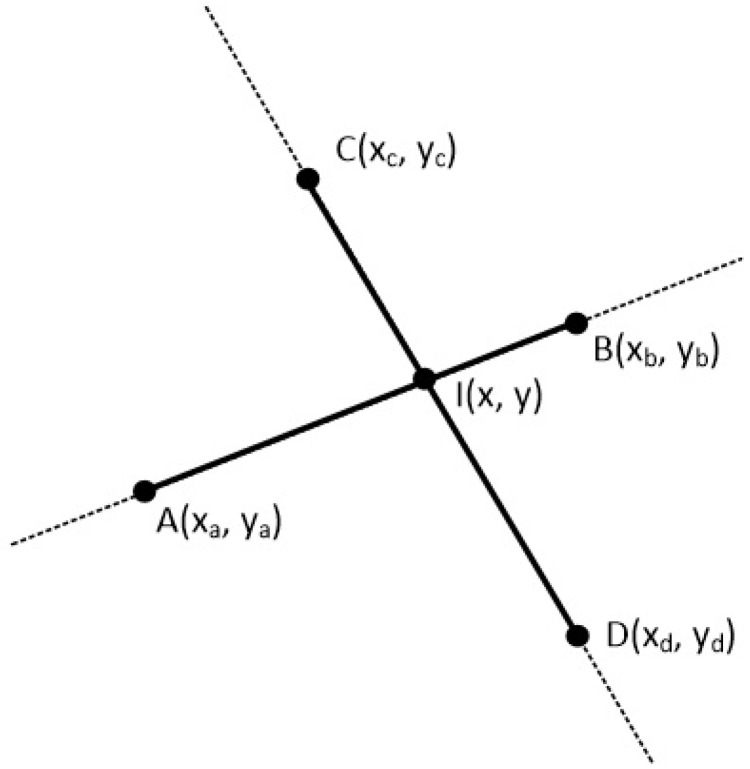
Two lines on a two-dimensional space.

The cross product may also be used for determining the intersection of two line segments. Let us assume that two points $A(x_{a},y_{a})$ and $B(x_{b},y_{b})$ define the line segment AB and two points $C(x_{c},y_{c})$ and $D(x_{d},y_{d})$ define the line segment CD on the two-dimensional space (see Figure 36).

The intersection of these two lines is a point I(x,y) with the following characteristics:

- The 2D cross product of point *I* with the line segment AB is zero.
- The 2D cross product of point *I* with the line segment CD is zero.

**Figure 37:**
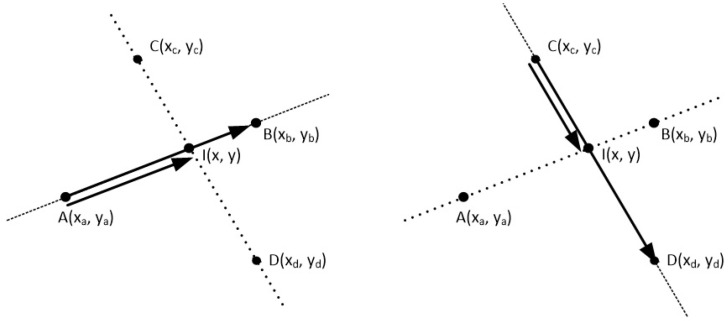
Cross product between the vectors AI and AB as well as between CI and CD is zero.

This is depicted better on Figure 37.

For the vectors AI and AB of Figure 37, the cross product is zero:

$$\vec{AI}\times \vec{AB}=0\Rightarrow$$

$$\left|\begin{array}{cc} \left(x-x_{a}\right) & \left(y-y_{a}\right)\\ \left(x_{b}-x_{a}\right) & \left(y_{b}-y_{a}\right) \end{array}\right|=0\Rightarrow$$

$$\left(x-x_{a}\right)\cdot \left(y_{b}-y_{a}\right)-\left(x_{b}-x_{a}\right)\cdot \left(y-y_{a}\right)=0\Rightarrow$$

$$x\cdot \left(y_{b}-y_{a}\right)-x_{a}\cdot \left(y_{b}-y_{a}\right)-y\cdot \left(x_{b}-x_{a}\right)+y_{a}\cdot \left(x_{b}-x_{a}\right)=0\Rightarrow$$

(5) $$\left(y_{b}-y_{a}\right)\cdot x-\left(x_{b}-x_{a}\right)\cdot y=x_{a}\cdot \left(y_{b}-y_{a}\right)-y_{a}\cdot \left(x_{b}-x_{a}\right)$$

For simplicity purposes, let us symbolize the next differences as

(6) $$dx_{1}=\left(x_{b}-x_{a}\right)\;\mathrm{and}\;dy_{1}=\left(y_{b}-y_{a}\right).$$

Combining (5) and (6) we obtain:

(7) $$dy_{1}\cdot x-dx_{1}\cdot y=x_{a}\cdot dy_{1}-y_{a}\cdot dx_{1}.$$

For the vectors $\vec{CI}$ and $\vec{CD}$ of Figure 37, the equation of the zero cross product would give:

(8) $$x\cdot \left(y_{d}-y_{c}\right)-y\cdot \left(x_{d}-x_{c}\right)=x_{c}\cdot \left(y_{d}-y_{c}\right)-y_{c}\cdot \left(x_{d}-x_{c}\right).$$

For reasons of simplicity, if we symbolize the next differences as

(9) $$dx_{2}=\left(x_{d}-x_{c}\right)\;\mathrm{and}\;dy_{2}=\left(y_{d}-y_{c}\right)$$

we obtain

(10) $$dy_{2}\cdot x-dx_{2}\cdot y=x_{c}\cdot dy_{2}-y_{c}\cdot dx_{2}$$

Equations (7) and (10) have two unknowns, so we can use their determinants for solving the system:

$$D=\left|\begin{array}{cc} dy_{1} & -dx_{1}\\ dy_{2} & -dx_{2} \end{array}\right|=dy_{2}\cdot dx_{1}-dy_{1}\cdot dx_{2}$$

$$\begin{array}{cc} DX & =\left|\begin{array}{cc} \left(x_{a}\cdot dy_{1}-y_{a}\cdot dx_{1}\right) & -dx_{1}\\ \left(x_{c}\cdot dy_{2}-y_{c}\cdot dx_{2}\right) & -dx_{2} \end{array}\right|\\ \; & =\left(x_{c}\cdot dy_{2}-y_{c}\cdot dx_{2}\right)\cdot dx_{1}-\left(x_{a}\cdot dy_{1}-y_{a}\cdot dx_{1}\right)\cdot dx_{2} \end{array}$$

$$\begin{array}{cc} DY & =\left|\begin{array}{cc} dy_{1} & \left(x_{a}\cdot dy_{1}-y_{a}\cdot dx_{1}\right)\\ dy_{2} & \left(x_{c}\cdot dy_{2}-y_{c}\cdot dx_{2}\right) \end{array}\right|\\ \; & =\left(x_{c}\cdot dy_{2}-y_{c}\cdot dx_{2}\right)\cdot dy_{1}-\left(x_{a}\cdot dy_{1}-y_{a}\cdot dx_{1}\right)\cdot dy_{2} \end{array}$$

Solving for *x* and *y*:

(11) $$x=\frac{DX}{D}=\frac{\left(x_{c}\cdot dy_{2}-y_{c}\cdot dx_{2}\right)\cdot dx_{1}-\left(x_{a}\cdot dy_{1}-y_{a}\cdot dx_{1}\right)\cdot dx_{2}}{dy_{2}\cdot dx_{1}-dy_{1}\cdot dx_{2}}$$

(12) $$y=\frac{DY}{D}=\frac{\left(x_{c}\cdot dy_{2}-y_{c}\cdot dx_{2}\right)\cdot dy_{1}-\left(x_{a}\cdot dy_{1}-y_{a}\cdot dx_{1}\right)\cdot dy_{2}}{dy_{2}\cdot dx_{1}-dy_{1}\cdot dx_{2}}.$$

We can create a function in pseudo-code (C++ based) that accepts two pairs of points, where each pair represents a line segment, and returns the intersection point of these line segments. Of course, we also take advantage of the similarities between the Equations (11) and (12).

point intersection(point A, point B, point C, point D)

{

// calculate the intersection point between lines AB and CD

point d1 = {B.x - A.x, B.y - A.y};

point d2 = {D.x - C.x, D.y - C.y};

float n1 = C.x * d2.y - C.y * d2.x;

float n2 = A.x * d1.y - A.y * d1.x;

float n3 = 1/(d2.y * d1.x - d1.y * d2.x);

float x = (n1 * d1.x - n2 * d2.x) * n3;

float y = (n1 * d1.y - n2 * d2.y) * n3;

return {x, y};

}

### 7.3. Description of the Algorithm

**Figure 38:**
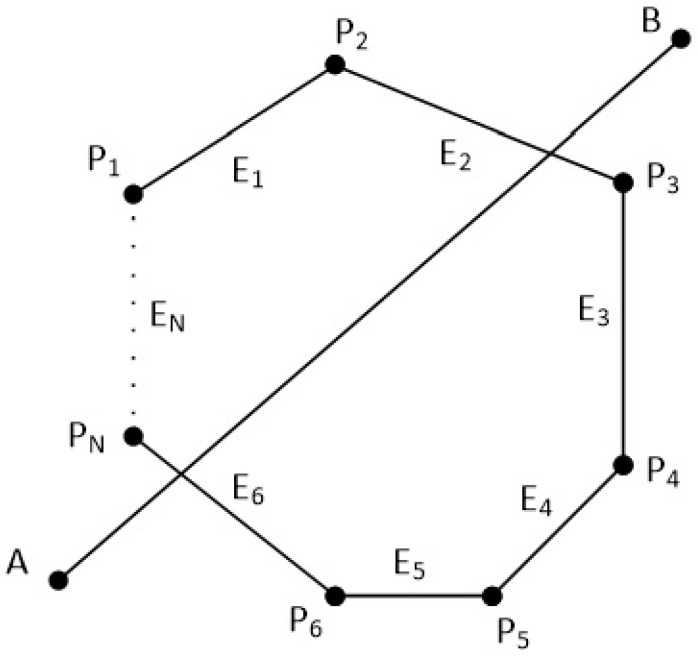
A convex polygon with *N* points and *N* edges and a line in the 2D space.

**Figure 39:**
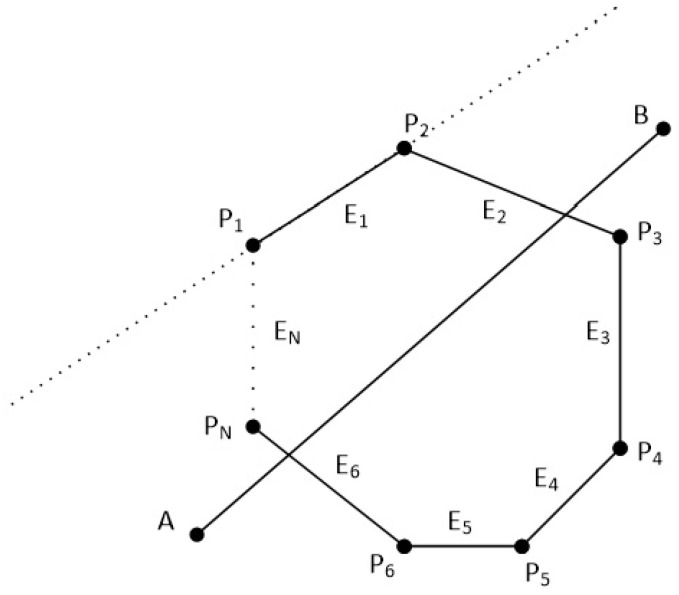
Checking if points *A* and *B* are left, right or on the edge $E_{1}$ of the polygon.

The clipping polygon has *N* vertices which are given in clockwise order. The first point of the polygon is $P_{1}\left(x_{1},y_{1}\right)$ and the last point is $P_{N}\left(x_{N},y_{N}\right)$. There are also *N* edges with names from $E_{1}$ to $E_{N}$. The line segment is defined by the points $A(x_{a},y_{a})$ and $B(x_{b},y_{b})$. Clockwise order means that as we go through the edges from $E_{1}$ to $E_{N}$, if a point is left of an edge then the 2D cross product is greater than zero, if a point is right of an edge then the 2D cross product is less than zero and if a point is on the edge then the 2D cross product is just zero (see Figure 38 and Figure 39).

### 7.4. Analysis

In order to clip the line, we have to go through each edge of the polygon in clockwise order and find where the points *A* and *B* reside compared to each edge. By residing, we mean that we have to determine, if the points *A* and *B* are to the left, to the right or on the edge (see Figure 39).

**Figure 40:**
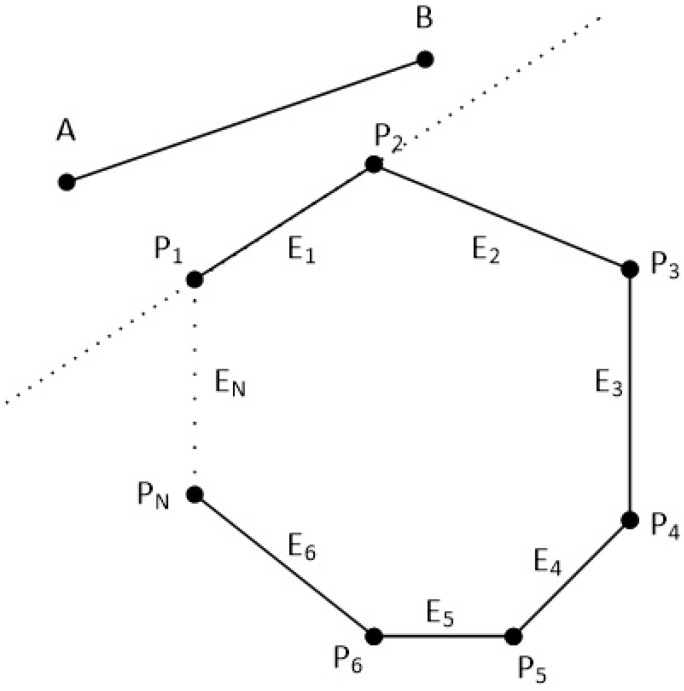
If points *A* and *B* are on the left side of an edge then the line is completely outside.

If both points *A* and *B* are to the left side of an edge then we reject the line as it is completely outside of the polygon, we draw nothing and the algorithm stops (see Figure 40).

**Figure 41:**
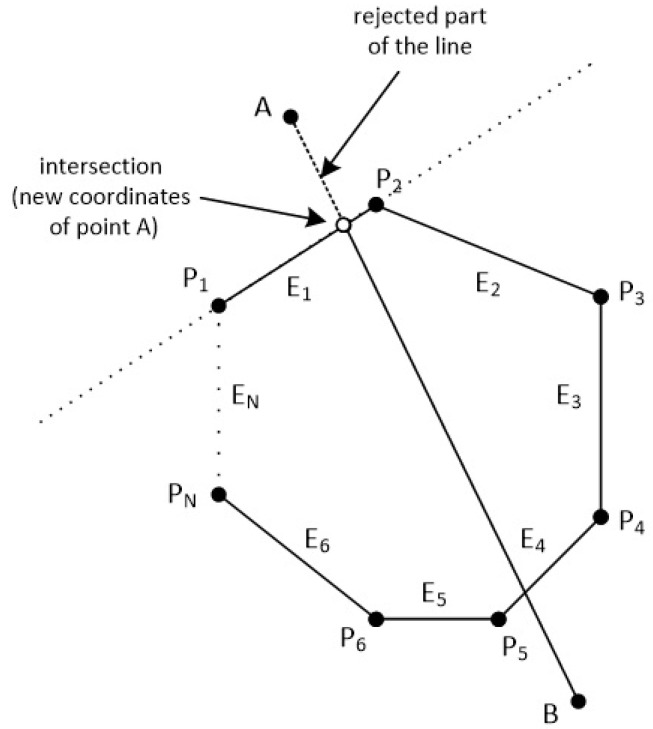
The intersection point replaces the point that it is outside the polygon.

If point *A* is left of the edge and point *B* is right of the edge or on the edge, we calculate the intersection point between the edge and the line segment AB. Then, we replace point *A* with the intersection point (see Figure 41).

Similarly, if point *B* is left of the edge and point *A* is right of the edge or on the edge, we calculate the intersection point between the edge and the line segment AB and we replace point *B* with the intersection point.

If points *A* and *B* are:

- Simultaneously right to the edge.
- One of them is on the edge and the other is right of the edge.

Then, we proceed to the next edge and repeat the same process or we stop if all of the *N* edges have been checked.

**Figure 42:**
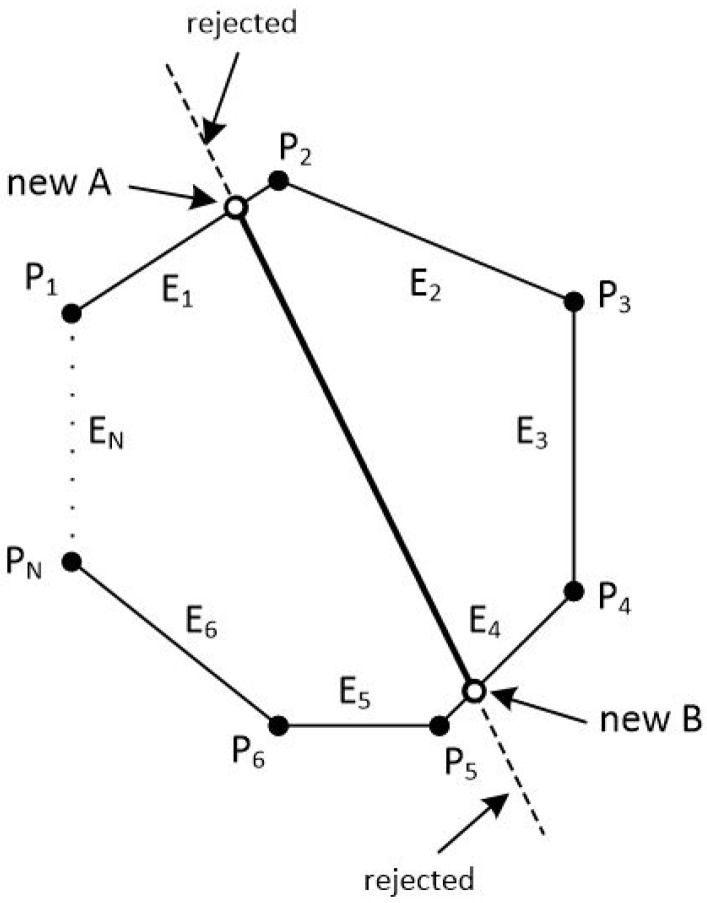
At the end, we draw a line segment from clipped point *A* to clipped point *B*.

At the end, we draw a line segment from clipped point *A* to clipped point *B* (see Figure 42).

### 7.5. The Steps of the Algorithm

1. Check against an edge of the polygon which is defined by the vertices $P_{i}\left(x_{i},y_{i}\right)$ and $P_{i+1}\left(x_{i+1},y_{i+1}\right)$ where the points $A(x_{a},y_{a})$ and $B(x_{b},y_{b})$ of the line reside. Each point may reside to the left of the edge, to the right of the edge or on the edge.
2. If both of the points are to the left of the edge then stop the algorithm and draw nothing. The line is completely outside the convex polygon.
3. If only point *A* is to the left of the edge then calculate the intersection point between the edge and the line. Replace the coordinates of point *A* with those coordinates of the intersection point and repeat from Step 1 with the next edge.
4. If only point *B* is to the left of the edge then calculate the intersection point between the edge and the line. Replace the coordinates of point *B* with those coordinates of the intersection point and repeat from Step 1 with the next edge.
5. If both points *A* and *B* are to the right of the edge or one of them is on the edge and the other is right of the edge then repeat from Step 1 with the next edge or stop if you have checked all *N* edges.
6. Draw the clipped line from point *A* to point *B*.

### 7.6. Pseudo-Code (C++ Based)

// INPUT : point A, point B, N, point polygon[N + 1] (last vertex is equal to first)

// OUTPUT : clipped line segment from point A to point B

float sideA, sideB;

bool draw = true;

for(int i = 0; i < N; i++)

{

// sideX > 0 --> LEFT

// sideX < 0 --> RIGHT

// sideX = 0 --> ON THE EDGE

sideA = cross_product(A, polygon[i], polygon[i + 1]);

sideB = cross_product(B, polygon[i], polygon[i + 1]);

if(sideA > 0 && sideB > 0)

{

// line is completely outside

draw = false;

break;

}

if(sideA > 0 && sideB <= 0)

// point A is outside, point B is inside polygon or on the edge

A = intersection(A, B, polygon[i], polygon[i + 1]);

else if(sideB > 0 && sideA <= 0)

// point B is outside, point A is inside polygon or on the edge

B = intersection(A, B, polygon[i], polygon[i + 1]);

}

if(draw)

draw_line(A, B);

## 8. Evaluation of All Line-Clipping against a Convex Polygon Algorithms

**Figure 43:**
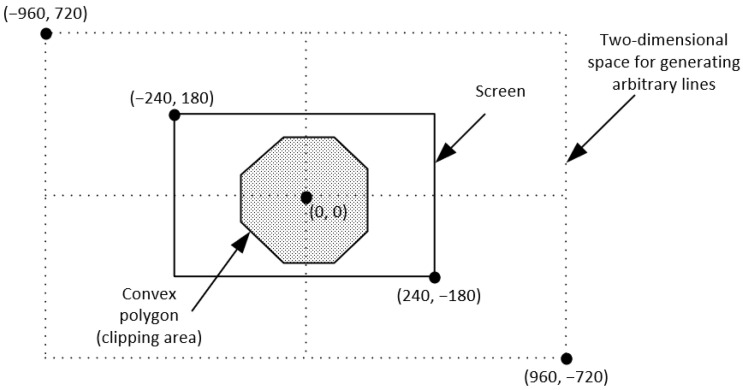
The two-dimensional space for generating arbitrary lines.

**Figure 44:**
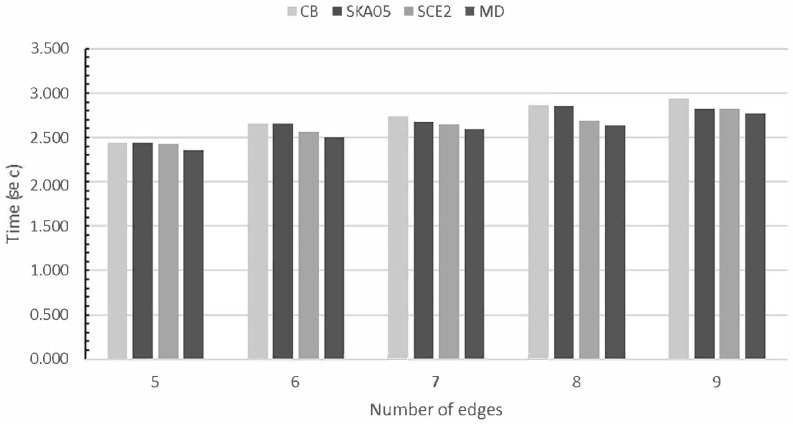
Average time of each algorithm when clipping 10 million lines against convex polygons with different number of edges (lower is better).

**Figure 45:**
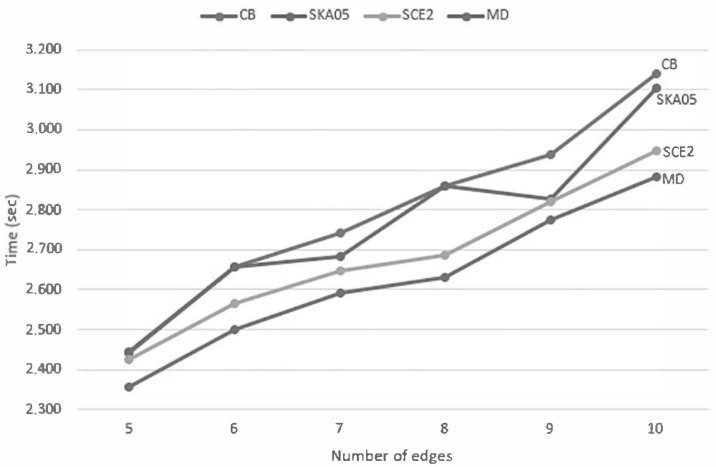
Speed graph of each algorithm when clipping 10 million lines against convex polygons with different number of edges (lower is better).

In order to determine the efficiency of all line clipping against convex polygon algorithms, we benchmarked them in the following way: Each one was creating 10,000,000 arbitrary lines in a two-dimensional space. The limits of this space were the points (−960,720) and (960,−720). The convex polygon (clipping area) was drawn somewhere inside our screen which had a resolution of 480 pixels width and 360 pixels height and with the center of the screen being the start of the axes X and Y (see Figure 43). The lines were randomly generated anywhere in the two-dimensional space and each algorithm had to clip and draw only the visible part of the lines inside the convex polygon. The total time that each algorithm needed to clip and draw these lines was recorded in every execution. The whole process was repeated 10 times and at the end, the average time was calculated; see Table 4, Figure 44 and Figure 45.

This process was repeated with many convex polygons with different vertices/edges. The hardware, as well as the software specifications for the evaluation, was: (a) Intel Core i7-9750H @2.60 Gz CPU, (b) RAM 16 GB, (c) NVIDIA GeForce RTX 2070/8 GB GPU, (d) Windows 10 Pro 64-bit operating system, (e) C++ with OpenGL/Freeglut under the Code::Blocks environment.

By using the formula

$$\frac{MD-other}{MD}\cdot 100$$

we can evaluate the speed of the *MD* algorithm in percent compared to the others. The next table shows this evaluation of speed; see Table 5.

So, comparing all the algorithms together we can conclude that the slowest of all is Cyrus–Beck. Skala 2005 is faster than Cyrus–Beck but not faster than the other two: S-clip E2 and *MD*. S-clip E2 performs very well but the performance of *MD* is high and steady in all cases.

## 9. Summary

The primary use of clipping in computer graphics is to remove objects, lines, or line segments that are outside the viewing pane and it is crucial. All objects that are not inside the field of view of the viewer have to be removed before generating the scene. For this reason, clipping is considered an important process. Clipping can be applied to objects like points, lines, polygons, curves, etc. Clipping a single point is rather easy, the clipping algorithm just accepts or rejects the point if its location is inside or outside the clipping window. However, when clipping a line or other objects, things are more complicated and more calculations have to be performed.

Many line-clipping algorithms in two dimensions have been developed over recent years. Each one has advantages and disadvantages. The computer programmer has to choose the suitable one according to his needs among a number of characteristics such as efficiency in calculations, the type of clipping area (rectangular, polygon or other), the type of mathematical approach (equations or vectors), whether the algorithm can be easily extended to other dimensions such as three dimensions and so on. This contribution briefly summarized common and uncommon line-clipping methods in 2D whereas it includes some of the latest research made by the authors.

## Figures and Tables

**Table 1:** Original values of the TAB-MASK table.

*c*	$c_{3}$	$c_{2}$	$c_{1}$	$c_{0}$	*TAB1*	*TAB2*	MASK
0	0	0	0	0	None	None	None
1	0	0	0	1	0	3	0100
2	0	0	1	0	0	1	0100
3	0	0	1	1	1	3	0010
4	0	1	0	0	1	2	0010
5	0	1	0	1	N/A	N/A	N/A
6	0	1	1	0	0	2	0100
7	0	1	1	1	2	3	1000
8	1	0	0	0	2	3	1000
9	1	0	0	1	0	2	0100
10	1	0	1	0	N/A	N/A	N/A
11	1	0	1	1	1	2	0010
12	1	1	0	0	1	3	0010
13	1	1	0	1	0	1	0100
14	1	1	1	0	0	3	0100
15	1	1	1	1	None	None	None

**Table 2:** Execution times of each algorithm when clipping 10,000,000 lines.

Exec.	CS	CB	LB	MS	NLN	SKA05	SCE2	KWC	*MD*
	(sec)	(sec)	(sec)	(sec)	(sec)	(sec)	(sec)	(sec)	(sec)
1	1.883	2.096	1.840	2.176	1.853	1.985	2.100	1.868	1.758
2	1.905	2.075	1.870	2.206	1.989	1.959	2.173	1.774	1.733
3	1.914	2.069	1.871	2.177	1.873	1.986	2.160	1.870	1.765
4	1.934	2.114	1.903	2.144	1.847	1.984	2.162	1.803	1.764
5	1.857	2.136	1.851	2.145	1.892	1.965	2.100	1.859	1.776
6	1.817	2.085	1.869	2.174	1.838	1.994	2.132	1.825	1.814
7	1.918	2.082	1.847	2.190	1.869	1.987	2.170	1.768	1.787
8	1.836	2.093	1.832	2.211	1.814	2.005	2.149	1.810	1.769
9	1.820	2.136	1.921	2.175	1.805	1.971	2.144	1.828	1.757
10	1.944	2.082	1.859	2.210	1.816	1.941	2.167	1.743	1.806
Avg:	1.883	2.097	1.866	2.181	1.860	1.978	2.146	1.815	1.773

**Table 3:** Percent that the *MD* algorithm is faster than the other algorithms.

The *MD* Algorithm Is % Faster Compared to							
**CS**	**CB**	**LB**	**MS**	**NLN**	**SKA05**	**SCE2**	**KWC**
6.20%	18.27%	5.27%	23.01%	4.89%	11.55%	21.03%	2.36%

**Table 4:** Average execution time of each algorithm when clipping 10 million lines against convex polygons with different numbers of edges.

*Number of Edges*	Cyrus–Beck (sec)	Skala 2005 (sec)	S-Clip E2 (sec)	Matthes–Drakopoulos (sec)
5	2.443	2.443	2.425	2.358
6	2.657	2.655	2.564	2.501
7	2.743	2.682	2.648	2.590
8	2.860	2.859	2.686	2.632
9	2.938	2.826	2.820	2.774
10	3.139	3.104	2.949	2.884

**Table 5:** Percent that the *MD* algorithm is faster than the other algorithms.

Edges	*MD* Algorithm Is Faster Compared to		
	Cyrus-Beck	Skala 2005	S-Clip E2
5	3.58%	3.61%	2.85%
6	6.21%	6.16%	2.50%
7	5.92%	3.54%	2.25%
8	8.65%	8.62%	2.07%
9	5.90%	1.87%	1.65%
10	8.84%	7.65%	2.26%

## Data Availability

Not applicable.
